# Supplementary material for: Transcriptomic Insights into Mechanisms of Early Seed Maturation in the Garden Pea (Pisum sativum L.)
Source: Cells. 2020 Mar 23;9(3):779. doi: 10.3390/cells9030779 (PMC7140803; doi:10.3390/cells9030779)
Supplement: Supplementary file 1 [file cells-09-00779-s001.zip › Supplementary materials/FileS3.zip!/FileS2/Hormones - Results/Summary Sheets - Samples/0verview.html]

Sample Summary of Hormones dataset


# Classification Overview

| Groups | Number of Samples |
| --- | --- |
|  |  |
| --- | --- |
| Sprint-2(10) | 4 |
| Sprint-2(20) | 4 |
| Zhewan-1(10) | 4 |
| Zhewan-1(25) | 4 |
| Zhongwan-6(10) | 4 |
| Zhongwan-6(25) | 4 |

# Sample Summary Sheets

For each sample a report sheet is created which summarizes the most
relevant information using the global and local perspective.
The global summary shows the ranked list of differentially expressed
genes for the whole sample, the ranked list of over- and underexpressed
gene sets after GSZ-overexpression analysis and the respective p-value
distributions.
The gene and gene set list are provided as tables.

| Sample Name | Group | Summary Sheet | Global Gene List | Gene Set List |
| --- | --- | --- | --- | --- |
| Sprint-2,10(1) | Sprint-2(10) | PDF | CSV | CSV |
| Sprint-2,10(2) | Sprint-2(10) | PDF | CSV | CSV |
| Sprint-2,10(3) | Sprint-2(10) | PDF | CSV | CSV |
| Sprint-2,10(4) | Sprint-2(10) | PDF | CSV | CSV |
| Sprint-2,20(1) | Sprint-2(20) | PDF | CSV | CSV |
| Sprint-2,20(2) | Sprint-2(20) | PDF | CSV | CSV |
| Sprint-2,20(3) | Sprint-2(20) | PDF | CSV | CSV |
| Sprint-2,20(4) | Sprint-2(20) | PDF | CSV | CSV |
| Zhewan-1,10(1) | Zhewan-1(10) | PDF | CSV | CSV |
| Zhewan-1,10(2) | Zhewan-1(10) | PDF | CSV | CSV |
| Zhewan-1,10(3) | Zhewan-1(10) | PDF | CSV | CSV |
| Zhewan-1,10(4) | Zhewan-1(10) | PDF | CSV | CSV |
| Zhewan-1,25(1) | Zhewan-1(25) | PDF | CSV | CSV |
| Zhewan-1,25(2) | Zhewan-1(25) | PDF | CSV | CSV |
| Zhewan-1,25(3) | Zhewan-1(25) | PDF | CSV | CSV |
| Zhewan-1,25(4) | Zhewan-1(25) | PDF | CSV | CSV |
| Zhongwan-6,10(1) | Zhongwan-6(10) | PDF | CSV | CSV |
| Zhongwan-6,10(2) | Zhongwan-6(10) | PDF | CSV | CSV |
| Zhongwan-6,10(3) | Zhongwan-6(10) | PDF | CSV | CSV |
| Zhongwan-6,10(4) | Zhongwan-6(10) | PDF | CSV | CSV |
| Zhongwan-6,25(1) | Zhongwan-6(25) | PDF | CSV | CSV |
| Zhongwan-6,25(2) | Zhongwan-6(25) | PDF | CSV | CSV |
| Zhongwan-6,25(3) | Zhongwan-6(25) | PDF | CSV | CSV |
| Zhongwan-6,25(4) | Zhongwan-6(25) | PDF | CSV | CSV |
